# Supplementary material for: MetaRibo-Seq measures translation in microbiomes
Source: Nat Commun. 2020 Jun 29;11:3268. doi: 10.1038/s41467-020-17081-z (PMC7324362; doi:10.1038/s41467-020-17081-z)
Supplement: Supplementary file 10 — Supplementary Data 7 [file 41467_2020_17081_MOESM10_ESM.zip › File2/Confidence_VeryHigh_Taxonomy/324358_out.krona.html]

Javascript must be enabled to view this page.

members
magnitude
magnitudeUnassigned
count
unassigned
taxon
rank

324358\_out

8

2
superkingdom
8

phylum
1239
8

186801
class
8

8
order
186802

31979
family
8

8
1485
genus

4
species
59620

SRS019068\_contig\_number\_27987SRS097920\_contig\_number\_31702SRS143148\_contig\_number\_25662SRS148784\_contig\_number\_8186


SRS019496\_contig\_number\_2874SRS024331\_contig\_number\_36799SRS147271\_contig\_number\_24878SRS893383\_contig\_number\_9420
2293014
species
4
